# Pressure‐dependent growth controls 3D architecture of *Pseudomonas putida* microcolonies

**DOI:** 10.1111/1758-2229.13182

**Published:** 2023-05-25

**Authors:** Juhyun Kim, Víctor de Lorenzo, Ángel Goñi‐Moreno

**Affiliations:** ^1^ School of Life Science BK21 FOUR KNU Creative BioResearch Group Kyungpook National University Daegu Republic of Korea; ^2^ Systems Biology Department Centro Nacional de Biotecnología (CNB‐CSIC) Cantoblanco‐Madrid Spain; ^3^ Centro de Biotecnología y Genómica de Plantas Universidad Politécnica de Madrid (UPM)‐Instituto Nacional de Investigación y Tecnología Agraria y Alimentaria (INIA/CSIC) Madrid Spain

## Abstract

Colony formation is key to many ecological and biotechnological processes. In its early stages, colony formation involves the concourse of a number of physical and biological parameters for generation of a distinct 3D structure—the specific influence of which remains unclear. We focused on a thus far neglected aspect of the process, specifically the consequences of the differential pressure experienced by cells in the middle of a colony versus that endured by bacteria located in the growing periphery. This feature was characterized experimentally in the soil bacterium *Pseudomonas putida*. Using an agent‐based model we recreated the growth of microcolonies in a scenario in which pressure was the only parameter affecting proliferation of cells. Simulations exposed that, due to constant collisions with other growing bacteria, cells have virtually no free space to move sideways, thereby delaying growth and boosting chances of overlapping on top of each other. This scenario was tested experimentally on agar surfaces. Comparison between experiments and simulations suggested that the inside/outside differential pressure determines growth, both timewise and in terms of spatial directions, eventually moulding colony shape. We thus argue that—at least in the case studied—mere physical pressure of growing cells suffices to explain key dynamics of colony formation.

## INTRODUCTION

Bacterial populations are ever‐present in natural and artificial environments (Flemming et al., 2016; Shirtliff et al., 2009), performing important tasks both beneficial (Hayat et al., 2010) and detrimental (García‐Betancur et al., 2017). This is why the analysis of bacterial communities and their physical architecture is a most active area or research (Flemming & Wuertz, 2019; Stoodley et al., 2002) that pursues both to update the current knowledge about their formation and performance (Sauer et al., 2022), and to develop the principles that allow for engineering new‐to‐Nature tasks in synthetic populations (Amos & Goni‐Moreno, 2018; Brenner et al., 2008; Jiang et al., 2022). To this end, mathematical models—in conjunction with experiments—are key tools since they help us interpret crucial dynamics behind population development that would be difficult (or entirely impossible) to obtain otherwise (Duran‐Nebreda et al., 2021; Dzianach et al., 2019; Espeso et al., 2016; Goñi‐Moreno et al., 2016). Due to the multiple population behaviours, types, roles, shapes and the many mathematical parameters involved in each of these, the study bacterial associations and communities is often tailored to a specific application—from bioproduction to human health (Gloag et al., 2020; Hays et al., 2015; Mukherjee & Cao, 2021; Pandit et al., 2020; Tran & Prindle, 2021). However, there are also developments aiming at establishing general‐purpose, often mechanical and biophysical, rules that can be applied in many different scenarios (Duvernoy et al., 2018; Martínez‐Calvo et al., 2022). Here we build on the latter framework and our previous work on motile populations (Espeso et al., 2016) to analyse the role of cell‐to‐cell pressure as the sole parameter affecting early colony formation.

The shape of the patterns arising from a growing bacterial colony, from fractals (Rudge et al., 2013) to radial expansion (Seminara et al., 2012), is the result of complex interactions between cells and their environment. Within this context, it is often challenging to infer colony performance even if it is fully known how a single cell behaves. Emergent properties such as nutrient gradients (Parsek & Tolker‐Nielsen, 2008) or functional clusters (van Gestel et al., 2015) turn colonies into somewhat single entities with features beyond its mere constituents—much like in ant colonies, despite the (of course) many differences.

From all the mathematical modelling approaches that are typically used to analyse colony formation, the one that is often used to capture emergent properties is agent‐based models (ABMs) (Nagarajan et al., 2022). ABMs are powerful frameworks that allow to decouple the performance of individual cells (i.e. each cell within the population is simulated independently) while describing space explicitly. The latter allow for modelling gradients (e.g. nutrients, signalling molecules, etc.) with local conditions depending on environmental parameters at specific spatial coordinates within the colony. Although computationally expensive—especially if intra‐cellular dynamics are simulated—there are recent efforts at parallelising the models so that exhaustive parameter exploration could be perform (Parry & Bithell, 2012). Altogether, the increasing activity in development of ABMs with different features provides a wide range of in silico tools to deconvolute intriguing experimental data. In this work, our in‐house ABM, called DiSCUS (Goni‐Moreno & Amos, 2015), was used to match experiments and predict colony performance.

In the course of our experiments with the soil bacteria *Pseudomonas putida* KT2440 we observed that an early‐stage colony growing on solid media always grew forming a 3D pyramid‐style structure. Although similar effects could be explained as a consequence of gradients in nutrient availability across the three‐dimensional population, the very small size of colonies suggested otherwise. As an alternative, the analysis presented here focuses on a pure mechanical aspect, namely the pressure that any cell is subject to as a consequence of growing surrounded by other growing bodies. The scenario that is tested below—both theoretically and experimentally—is that mechanical pressure affects cells growth in a 3D manner: cells at the centre would be the ones with highest pressure forces and, therefore, the first ones to look for an alternative to horizontal colony formation. Similar mechanical performance has been shown for *Escherichia coli* (Duvernoy et al., 2018; Grant et al., 2014), Vibrio cholerae (Yan et al., 2016), and Pseudomonas aeruginosa (Duvernoy et al., 2018). Here we expand this analysis to characterize *Pseudomonas putida* microcolonies, suggesting not only a differential pattering performance, but also differential growth rates. These results emphasize the weight of mere physical and environmental factors on biological phenomena often attributed to adaptive programmes.

## EXPERIMENTAL PROCEDURES

### Microscopy to study formations of bacteria microcolonies

To observe bacterial growth under physical constraints, we used P. putida KT2440‐GFP, expressing GFP constitutively (Espeso et al., 2016; Martinez‐Garcia et al., 2020), and the cell was routinely grown at 30°C. The reporter strain was cultured overnight in Luria–Bertani (LB) medium, then the bacterial cultures were 100‐fold dilute diluted in reduced concentration of LB medium (10%). When the sample reached exponential phase of growth (optical density at 600 nm [OD_600_] = 0.3), 1 μL of the culture was placed onto agarose pad including 10% of LB. To this end, we prepared dynamic concentration of agarose pad on a microscope slide glass (Young et al., 2012), followed by dropping the bacterial sample, and a coverslip was assembled on the slide. The growing cells on the agarose pad were recorded at 30 minutes time intervals using a Leica TCS SP5 confocal microscope equipped with a temperature control chamber. This approach allowed us to analyse 3D formation of cellular growth. We also cultivated the cell on the agarose pad overnight, and the growth formation in 2D was explored using an Olympus BX61 instrument. Green fluorescent signals were obtained with filters carrying an excitation laser (488 nm) built in both microscopes. Further image analysis was carried out using Fiji software.

### Simulations

All simulations were performed using the agent‐based software DiSCUS (Goñi‐Moreno et al., 2013; Goñi‐Moreno et al., 2019; Goni‐Moreno & Amos, 2015), which uses the physics engine *pymunk* to resolve collisions between rigid bodies. For the simulations shown in Figure 2 there is a vertical force emulating gravity so simulated cells grow in a vertical 2D environment to match confocal vertical planes. This force is removed in the simulations shown in Figure 3, so cells are allowed to form a circular‐like colony visualized from the top. The overlap of bodies takes place when growth is faster than the collision solver and, as a result, cells share the same space in the *z* axis.

## RESULTS AND DISCUSSION

### *3D formation in* P. putida *colonies*

**FIGURE 1:**
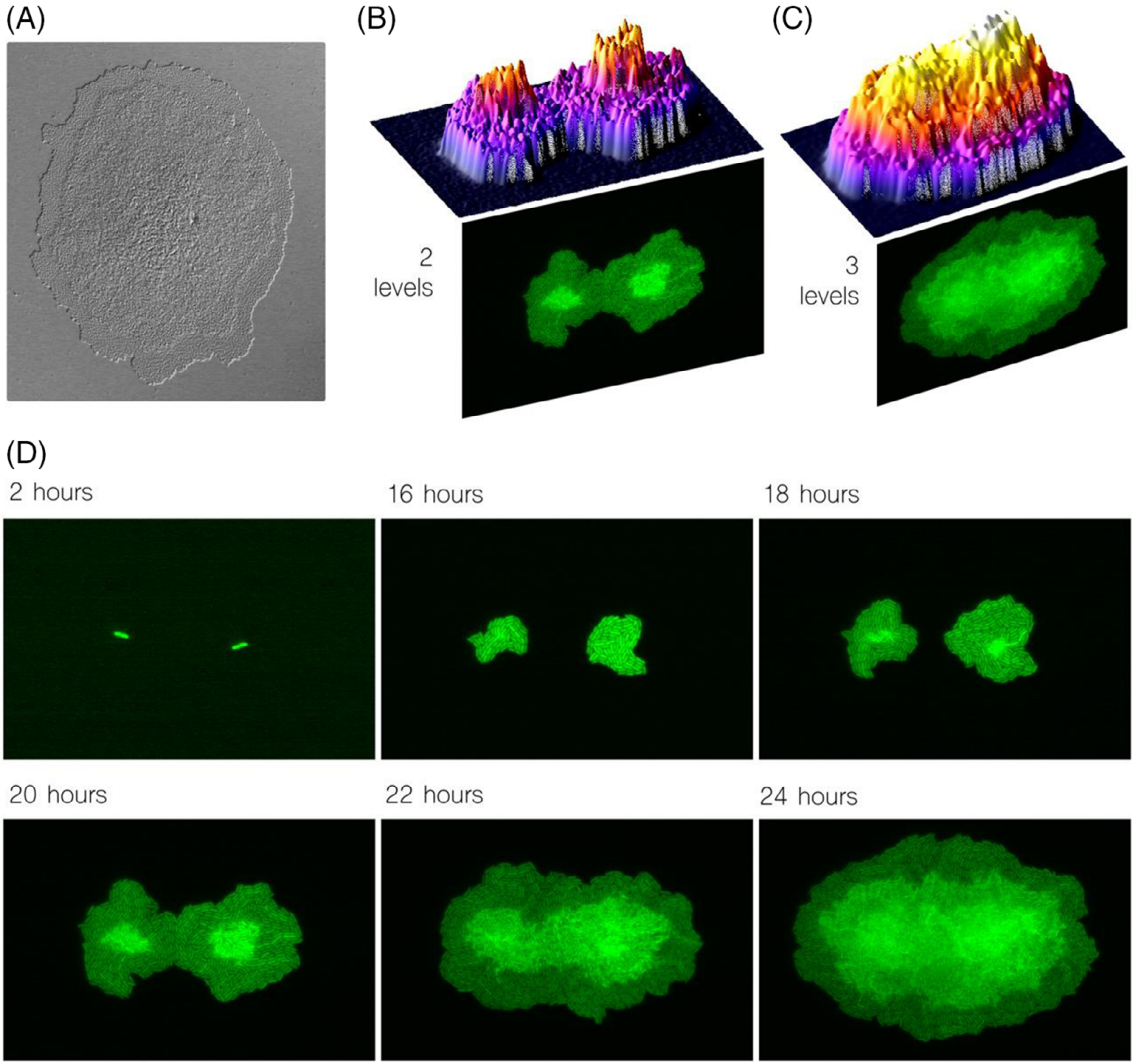
3D formation of *Pseudomonas putida* KT2440 microcolonies. (A) Microscopy image of a 2‐day colony (starting from a single cell), where different 3D levels are clearly visible: a 1‐cell deep outer ring, a 2‐cell deep middle ring, and an inner structure where height increases till the central point. (B,C) 3D surface maps of a colony after 20 h (B) with only two layers, and the same colony after 24 h (C) with the central structure. (D) Time‐lapse microscopy of the same colony from 2 h (first cellular division in two sub‐colonies) to 24 h. As observed, the sub‐colonies grow in perfect 2D shape till 18 h, where cells in the middle overlap on a second layer.

In experiments with enough nutrients, ideal temperature conditions and stress‐free environments, the bacterium *Pseudomonas putida* grows initially forming a 2D microcolony—a monolayer. When a specific threshold is reached, the system gets unbalanced, and a 3D structure starts to emerge to finally form a pyramid‐like arrangement (Figure 1A) where the centre of the colony is of a certain height, and the outer ring remains 1‐cell deep. The nature of that threshold and the causes of moving beyond it were the starting questions that motivated this work.

Despite the colony of Figure 1A showed a rather chaotic central ring where cells mount on top of each other building a gradual silhouette, early colonies (i.e. at initial stages in their development) showed a discrete system of layers (Figure 1B,C). Indeed, two distinct layers (1‐cell and 2‐cell deep) were distinguishable after only 20 h (starting from a single growing cell) and three layers (1‐cell, 2‐cell and 3‐cell deep) after 24 h in the same experiment, as observed by microscopy profiles. The most remarkable feature in Figure 1B,C is that the initial outer ring when the two colonies where separated remained coherent and homogeneous (i.e. all area of the rings performed the same; a monolayer in this case) till the two colonies met. At that moment the homogeneity was broken since the conditions for the cells of the outer ring at the intersection of two colonies were drastically changed. Bacteria in the confronting edges were suddenly trapped at the middle of two growing colonies, which presumably increased pressure to the same levels than in the middle of each population. This suggests pressure itself—and not nutrient availability—is responsible for 3D patter formation.

In Figure 1D, the same colony is shown at different stages, from 2 h after inoculation (when the first division happened) until 24 h. The second layer started to emerge at 18 h at the centre of each colony, and after 22 h at the intersection of both. Also, at 22 h the third layer started to emerge at the same spot than the second layer did.

### Measuring colony height and model matching

**FIGURE 2:**
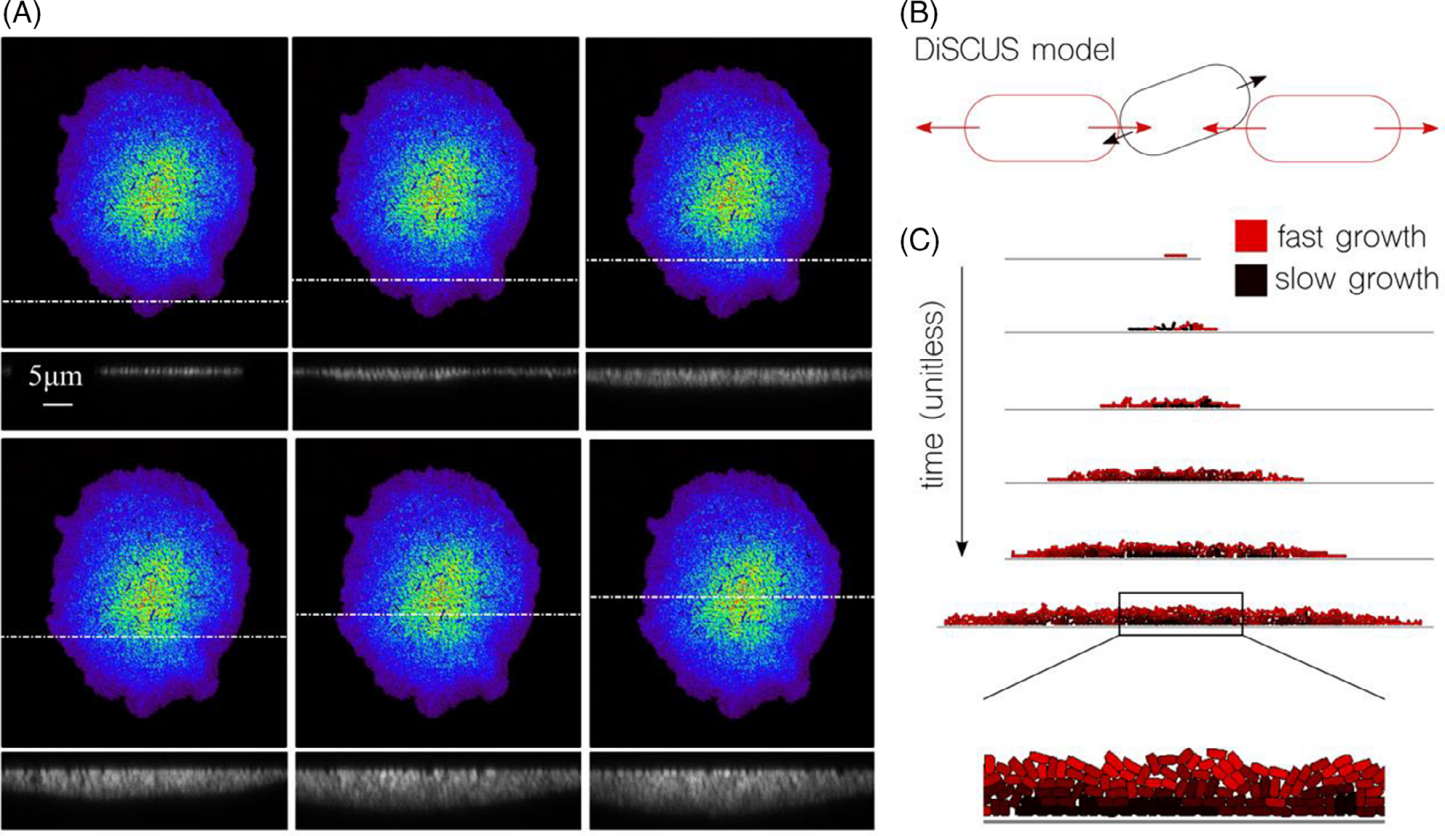
Height of a 3D colony at different locations and simulation of a 2D section. (A) Confocal microscopy analysis of a 3D Pseudomonas putida KT2440 colony, where the horizontal while line specifies the measuring section in each panel. As observed, the outer ring corresponds to a single cell layer. (B) The model, based on the DiSCUS software, defines growing rod‐shape objects whose expansion is asynchronous (using both poles at different times) and uses asymmetric division (random angles). Cells cannot grow sideways if they find strong pressure from other objects, so they build overlapping layers. (C) A 2D simulation where cells arrive at the experimentally observed structure.

We measured colonies using confocal microscopy (see Experimental procedures for details) in order to have quantitative values for the depth of each layer. The results of Figure 2A show the same colony as in Figure 1A but characterized using absolute units (μm) at different linear sections within the population. The colour scale also provides a qualitative indication of the different layers. The first section cut corresponds to a monolayer and can be used as a reference for the following. It can be observed that the height of the monolayer was approximately doubled at the second layer, and multiplied by 3 at the next to finally correlate to 5–6 times as high in the middle of the colony. The discrete performance of the patterning can also be observed, as shown by the detail highlighted in the second image of Figure 2A. Here the section measured by confocal microscopy crossed two layers horizontally, the first and the second ones. The vertical profile of the section showed that the transition from the first to the second follows a discrete jump, meaning the layers are perfectly defined by one or two cells deep. However, that discreteness was not observed at the middle of the colony, where the section profiles showed gradual increments to finally build the aforementioned pyramid‐style structure. The qualitative information provided by the colouring of the layers agrees with this observation—the blue and purple cells corresponding to the first and second layers (respectively) are clearly separated, while the green, yellow and red are already mixed.

In order to get a deeper understanding of the mechanistic origins of the previous observations, we used an agent‐based model (DiSCUS) where cells were modelled independently (each had independent growth) and collisions between the objects (i.e. simulated cells) were resolved dynamically using a physics engine (see Experimental procedures for more details). Cellular elongation was modelled asynchronously (i.e. growing from both poles at random times) and division with asymmetry (i.e. after division objects had slightly random variations in angles). After the simulation of colonies growing from one single object, we observed that objects trapped in the middle of a dense population had more collisions to resolve. This is to say, there were more cell‐to‐cell contacts within the central region of a radially growing colony. We then added pressure‐dependent growth to the objects so that elongation steps where delayed (but not cancelled entirely) if objects had to resolve many collisions, that is, cells were being pushed by other surrounding bacteria. As a result, object trapped in central regions showed a slower growth and—crucially—tended to move towards collision‐free space (Figure 2B).

What is shown in Figure 2C is a two‐dimensional vertical simulation which results are comparable to the experimental observations of Figure 2A. We simulated a cell growing on a solid surface in a section‐like environment, since we were simulating the confocal microscopy results. Two main conclusions were inferred from comparing simulations against experiments that helped confirm the pressure‐dependent hypothesis behind pattern formation. The first one is that layering happens in a discrete fashion as can be observed at the far left and right extremes of the simulated colony, where a monolayer paves the way for a 2‐cell deep layer. However, in the central area of the colony the transition between layers is gradual—which agrees with experimental observations—and finally builds the pyramid‐like structure. The second conclusion is that cells trapped in the central region, and below other layers, grew slower than the cells with free space available (colour difference in Figure 2C). Unlike the previous feature (layer height) the age of the cells could not be obtained, nor inferred, from our experiments. Indeed, models can analyse a wider range of parameters than experiments. However, the addition of pressure‐dependent growth in the mathematical simulation gives as a consequence these differences in ageing within the colony. Since simulations match the experimental patterns, it is therefore suggested that this phenomenon is direct effect of this type of colony development.

### Relationship between pattern, cell age and pressure

**FIGURE 3:**
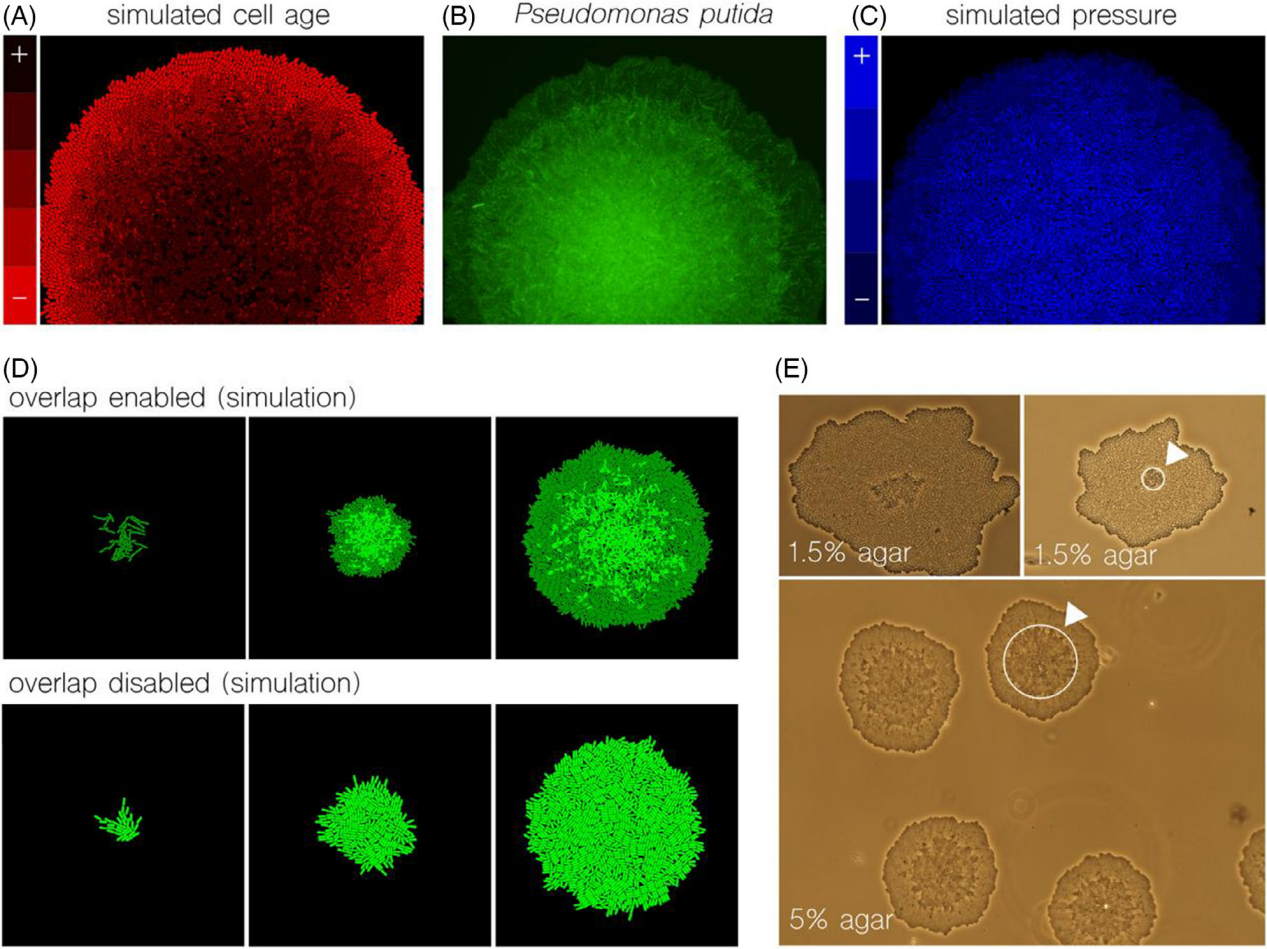
Implications and causes of early 3D formation. (A–C) Cells growing in a 3D microcolony are in a different physicochemical state depending on the layer they are in. A simulation highlighted the cells in the outer ring were younger (A) than in the centre, where age is equivalent to their generation (from the first division). The pattern emerging from the age‐dependent visualization matches the shape of the fluorescent rings in a *Pseudomonas putida* KT2440 microcolony (B). A similar pattern arose in a simulation where pressure was visualized (C), being the cells trapped at the centre the ones with more physical pressure on their bodies. Here, age and pressure are inversely correlated. (D) The causes of the overlapping behaviour were modulated in simulations. While a standard simulation allowed cells to overlap (top), a surface that allowed for easier movements generated an ideal monolayer (meaning bodies had longer time to resolve collisions). (E) Experimental tuning of 3D formation. The % of agar in the media correlated to how long it took the overlapping behaviour to emerge (the lower %, the easier sideways movement). When % agar was set to 5% (high), 3D formation occurs at an earlier stage. Our hypothesis is that a more solid surface generates more frictions in the 2D starting colony, so more pressure at the inner ring. This performance matches simulations in (D).

As pointed out before, simulations that were able to match experimental observations suggested that the differentiation in terms of cellular age is a needed consequence of 3D pattern formation. We showed direct visual comparison of a radially growing colony in Figure 3A–C to highlight the correlation between the three features. Figure 3A shows a simulation where the colour scale represents cellular age, measured in terms of the time it takes for a single object to divide into two—the longer it takes, the older the object is. Here the population showed a clearly radial age differentiation, being the central region where older cells accumulated. Also, the age differentiation pointed at the discreteness of the layering at the outer areas of the colony, where colours are physically separated into distinct regions. This simulated parameter (age) correlated well to the fluorescent output of a growing *Pseudomonas putida* population (Figure 3B) in terms of differentiated areas and overall patterning. Also, the correlation stays strong when matching against a simulation where the measured signal was pressure (Figure 3C). This suggested that cellular age (or growth times), layered‐based patterns and pressure across the microcolony are three correlated parameters.

### Surface strength affecting 3D formation

We then analysed a parameter—the strength of the surface—that was predicted to influence 3D pattern formation according to simulations. As it was pointed out before, the model gave as a result the overlapping behaviour between cellular objects when there was not enough time to resolve collisions. That is to say, cells that were surrounded by other growing bodies pushing in all directions—being each ‘push’ a collision—started to move on top of each other. As can be seen in the standard simulation of Figure 3D (top) the cells at the centre, where pushing/pressure forces were maximized, broke the initial monolayer (light green in the figure). We then wondered if we could force the simulation to generate an infinite monolayer that could not be broken at any time. The increase of the time it takes to resolve collisions worked well for this purpose as can be seen in Figure 3D (bottom). In this simulation the cells are never trapped within pressure forces, since each object have enough time to resolve every collision and move accordingly. This simulated (extremely) slow growth is not realistic since cells will reach division while—but not after—handling pressure. Experimentally we attempted, successfully, to recreate this phenomenon by decreasing the stiffness of the surface where bacteria were growing on (Figure 3E).

To this end, we reduced the concentration of agar in the media used to grow the colony. On the course of these experiments, we observed that a lower agar concentration (represented by its percentage) resulted in a bigger monolayer (Figure 3E, agar at 1.5%), that is, cells at the central region did not overlap until the microcolony had grown more than in previous occasions. According to the modelling outcome this suggests that cells were moving on a low‐friction surface, thus finding easier displacement due to pressure/pushing forces caused by other microbes. Concurrently, we run an experiment where agar concentration was increased (Figure 3E, agar at 5%) and the formation of the second layer took place at an earlier stage in the development of the microcolony. Following again the conclusions of the model, this latter case suggests that cells that were pushed by others found increase friction on the surface causing limited (and not enough) displacements.

## CONCLUSION

The study above deals with the physics of microcolony formation in growing *Pseudomonas putida* (Chavarría et al., 2016; Kim et al., 2019), in particular the characterization of their emerging three‐dimensional architectures prior to involvement of any biological program. To this end, we adopted a mathematical model that predicted specific scenarios of colony development, which were then tested experimentally with success. Whether such early microcolonies evolve afterwards for forming a biofilm depends on a number of environmental and endogenous cues that bacteria may process at a later stage—beyond the time frame applicable in our case. In any case, we entertain that the earlier pressure‐dependent patterning and cellular age differentiation endured by the forerunner cells when start growing on a flat surface, has a sort of foundational effect for whatever morphological route the population follows. How can this happen?

The way patterns were observed to emerge is a direct consequence of the pressure forces that cells were being exposed to. This pressure was defined in this study as the pushing forces that were physically applied on each cellular body. Cellular expansion was affected by a phenomenon that we termed *pressure‐dependent growth*. While cells at the centre of the microcolony were all squeezed together, those at the outer ring had different—and opposite—physical conditions. As a result, the microcolony forms consecutive layers (one on top of each other) as long as pressure on cells move above a certain threshold. We tested this phenomenon, showing computational simulations as well as experimental results. One intriguing conclusion is that the physical conditions of the environment where the microcolony grows become the major determinant of its later development (Martínez‐Calvo et al., 2022). Given that the topology and the granularity of environmental surfaces would be way more complex than the flat substratum used for this analysis, one can expect a considerable diversification of pressures and ages of bacteria at a given site. These circumstances can surely result in a multiplicity of 3D arrangements and shapes of the grown population which are largely fostered by purely physical forces, instead of any present genetically encoded program.

## AUTHOR CONTRIBUTIONS

**Juhyun Kim:** Formal analysis (equal); investigation (equal); methodology (equal); writing – review and editing (equal). **Victor de Lorenzo:** Formal analysis (equal); funding acquisition (equal); writing – review and editing (equal). **Ángel Goñi‐Moreno:** Formal analysis (equal); funding acquisition (equal); investigation (equal); methodology (equal); writing – original draft (equal); writing – review and editing (equal).

## CONFLICT OF INTEREST STATEMENT

The authors declare no competing financial or non‐financial interests.

## Data Availability

The raw confocal and fluorescence microscopy files used in this analysis, along with extra images, can be found at https://doi.org/10.21950/ZAX7YW. The code for the simulations can be found at https://github.com/Biocomputation-CBGP/DiSCUS-PG with information on how to reproduce the simulations shown in the figures of this work. All data is openly accessible.
